# NFκB-Mediated Expression of Phosphoinositide 3-Kinase δ Is Critical for Mesenchymal Transition in Retinal Pigment Epithelial Cells

**DOI:** 10.3390/cells12020207

**Published:** 2023-01-04

**Authors:** Haote Han, Yanhui Yang, Zhuo Han, Luping Wang, Lijun Dong, Hui Qi, Bing Liu, Jingkui Tian, Bart Vanhaesebroeck, Andrius Kazlauskas, Guoming Zhang, Shaochong Zhang, Hetian Lei

**Affiliations:** 1 Institute of Basic Medicine and Cancer, Cancer Hospital of the University of Chinese Academy of Sciences (Zhejiang Cancer Hospital), Hangzhou 100864, China; 2 Schepens Eye Research Institute of Massachusetts Eye and Ear, Boston, MA 02114, USA; 3 Department of Ophthalmology, Harvard Medical School, Boston, MA 02115, USA; 4 Ningxia Key Laboratory of Prevention and Control of Common Infectious Diseases, The School of Basic Medical Sciences, Ningxia Medical University, Yinchuan 750101, China; 5 Shenzhen Eye Hospital, Jinan University, Shenzhen Eye Institute, Shenzhen 518000, China; 6 Guangzhou Women and Children’s Medical Center, Guangzhou Medical University, Guangzhou 510180, China; 7 Cancer Institute, University College London, London WC1E 6BT, UK; 8 Department of Ophthalmology and Visual Sciences, Department of Physiology and Biophysics, University of Illinois at Chicago, Chicago, IL 60607, USA

**Keywords:** TGF-β2, PI3Kδ, NFκB/p65, CRISPR/Cas9, RPE, EMT, PVR

## Abstract

Epithelial mesenchymal transition (EMT) plays a vital role in a variety of human diseases including proliferative vitreoretinopathy (PVR), in which retinal pigment epithelial (RPE) cells play a key part. Transcriptomic analysis showed that the phosphoinositide 3-kinase (PI3K)/Akt signaling pathway was up-regulated in human RPE cells upon treatment with transforming growth factor (TGF)-β2, a multifunctional cytokine associated with clinical PVR. Stimulation of human RPE cells with TGF-β2 induced expression of p110δ (the catalytic subunit of PI3Kδ) and activation of NFκB/p65. CRISPR-Cas9-mediated depletion of p110δ or NFκB/p65 suppressed TGF-β2-induced fibronectin expression and activation of Akt as well as migration of these cells. Intriguingly, abrogating expression of NFκB/p65 also blocked TGF-β2-induced expression of p110δ, and luciferase reporter assay indicated that TGF-β2 induced NFκB/p65 binding to the promoter of the *PIK3CD* that encodes p110δ. These data reveal that NFκB/p65-mediated expression of PI3Kδ is essential in human RPE cells for TGF-β2-induced EMT, uncovering hindrance of TGF-β2-induced expression of p110δ as a novel approach to inhibit PVR.

## 1. Introduction

Epithelial mesenchymal transition (EMT), a process by which epithelial cells lose their cell polarity and gain mesenchymal properties, is essential for animal development and wound healing. EMT also plays a critical role in a variety of human diseases including organ fibrosis, cancer metastasis and proliferative vitreoretinopathy (PVR). PVR, a fibrotic eye disease, develops in 8–10% of patients who undergo retinal surgery to correct rhegmatogenous retinal detachment (RRD) [1,2,3,4,5] and in 40–60% of those with open-globe injury [6,7,8,9,10,11,12,13]. There are an estimated 55,000 individuals experiencing RRD in the United States annually [14] and approximately 203,000 cases of open-globe injury worldwide each year [6,10,15]. Currently, repeat surgery is the only option for treating PVR [16,17,18]; however, the outcome of the surgery to restore vision is poor due to the retinal damage resulting from recurrent detachment and the PVR process itself [19]. In addition, adjuvant pharmacotherapies that have been evaluated thus far have neither blocked the formation of PVR nor consistently reduced the rates of retinal re-detachment secondary to PVR [18,19,20,21,22,23]. It is therefore important to understand the molecular mechanisms underlying PVR.

A major characteristic of PVR is the formation of epi- or sub-retinal membranes (ERMs), which consist of an extracellular matrix and cells including retinal pigment epithelial (RPE) cells, Muller’s glia, fibroblasts and macrophages [5,21]. RPE cells are considered to be important and central players in the pathogenesis of PVR [5,24]. When the retina tears or is detached, quiescent retinal cells such as RPE cells undergo a series of responses including proliferation, EMT, migration and secretion of extracellular proteins [5,25,26] to form ERMs, whose contraction causes retinal detachment [5,21].

Phosphoinositide 3-kinases (PI3Ks) play a central role in experimental PVR [27,28,29]. Key agonist-sensitive PI3Ks are the receptor-regulated class IA PI3Ks that consist of a regulatory p85 subunit in complex with a p110 catalytic subunit (p110α, -β or -δ) [30,31]. p110δ expression is highly enriched in white blood cells [32] and present at a low level in non-leukocytes where its levels can be enhanced by stimuli such as tumor necrosis factor (TNF)α [30] or high glucose [33] in human vascular endothelial cells (ECs).

TNFα mediates most of its transcriptional effects via the NFκB family of transcription factors (TFs). In the nucleus, NFκB dimers bind to target DNA elements and activate transcription of genes encoding proteins involved in immune or inflammation responses and cell growth control. NFκB/p65 has been shown to promote transcription of p110δ in human non-leukocytes [30]. We recently showed that PI3Kδ contributes to the vitreous-induced activation of Akt and murine double minute (Mdm)2, which plays an essential role in experimental PVR [34]. However, the upstream regulatory mechanism underlying the observed increase in p110δ levels has remained elusive.

Based on prior evidence [30,31,32,33,34], we hypothesized that vitreal factors including TGF-β2 induced NFκB/p65-mediated p110δ expression in RPE cells, triggering cellular responses such as EMT. In this study, we executed a series of experiments to test this hypothesis.

## 2. Materials and Methods

### 2.1. Major Reagents

TGF-β2 was purchased from R&D Systems (Minneapolis, MN, USA). Primary antibodies against p-Ser473 Akt, Akt, NF-κB, pSer536 NF-κB, Snail, p110δ, fibronectin, pan-keratin, N-cadherin and β-catenin were purchased from Cell Signaling Technology (Danvers, MA, USA), and β-actin was ordered from Santa Cruz Biotechnology (Santa Cruz, CA, USA). Horseradish peroxidase-conjugated mouse anti-rabbit IgG and goat anti-mouse IgG were ordered from Santa Cruz Biotechnology. Alexa fluorescence-488-conjugated mouse anti-rabbit IgG and Alexa fluorescence-549-conjugated mouse anti-rabbit IgG were ordered from Vector laboratories Inc. (Burlingame, CA, USA). Enhanced chemiluminescent substrate to detect horseradish peroxidase was purchased from Thermo Fisher Scientific (Waltham, MA, USA).

ARPE-19 cells (American Type Culture Collection: ATCC, Manassas, VA, USA), a human RPE cell line, were cultured in Dulbecco’s modified Eagle’s medium/nutrient mixture (DMEM/F-12, Invitrogen) supplemented with 10% fetal bovine serum (FBS). ARPE-19 cells with a *lacZ* or PK2 single guide (sg) RNA were generated as described previously [34,35]. sgRNA (named PK2, AGAGCGGCTCATACTGGGCG) from exon 4 in the human genomic *PIK3CD* locus was able to guide SpCas9 to cleave the locus at its expected site, resulting in 90% depletion of p110δ in ARPE-19 cells analyzed by Western blotting [34,35]; human embryonic kidney (HEK) 293 T cells (HEK293, containing SV40 T-antigen) from ATCC, were cultured in DMEM (4.5 g/L D-glucose) with 10% FBS. The culture medium to produce lentivirus by HEK293T cells was DMEM (4.5 g/L D-glucose) supplemented with 20% FBS. All cells were cultured at 37 °C in a humidified 5% CO_2_ atmosphere.

### 2.2. RNA Sequencing

When cells had grown to 90% confluence in 10 cm dishes, they were treated with 10 ng/mL TGF-β2 for 48 h (h). Subsequently, total RNA was extracted using TriZol reagent (Thermo Fisher Scientific) according to the manufacturer’s procedure. Differential gene expression analysis was performed by DESeq2 software (www.bioconductor.org (accessed on 24 November 2021)) between two different groups (and by edge R between two samples). The genes with the parameter of false discovery rate (FDR) below 0.05 and absolute log2 fold change ≥1.1 were considered differentially expressed genes. Differentially expressed genes were then subjected to enrichment analysis of GO functions and KEGG pathways.

### 2.3. DNA Constructs

The 20-nt target DNA sequences preceding a 5′-NGG PAM sequence at exon 6 in the human genomic *RELA* locus (NG_029971.1) were selected to generate sgRNA for SpCas9 targets using the CRISPR design website (http://chopchop.cbu.uib.noorg (accessed on 7 September 2020)). The control sgRNA sequence (5′-TGCGAATACGCCCACGCGATGGG-3′) was designed to target the *lacZ* gene from *Escherichia coli*. The lentiCRISPR v2 vector was purchased from Addgene (catalog #52961) [34].

To express SpGuides in the targeted cells, the top oligonucleotides 5′-CACCG-20-nt (target *RELA* DNA sequence p65 (ACTACGACCTGAATGCTGTG) or the *lacZ* sgRNA sequence)-3′ and the bottom oligonucleotides 5′-AAAC-20-nt (20-nt: complementary target *RELA* DNA sequences or *lacZ* sgRNA sequence)-C-3′ were annealed and cloned into the lentiCRISPR v2 vector by *BsmBI* (New England Biolabs, Boston, MA, USA), respectively. All clones were confirmed by DNA sequencing using primer 5′-GGACTATCATATGCTTACCG-3′ from a sequence of the U6 promoter that drives expression of sgRNAs. DNA synthesis and sequencing were performed by Massachusetts General Hospital DNA Core Facility (Cambridge, MA, USA).

### 2.4. Lentivirus Production

Lentivirus was produced as described previously [34]. Briefly, the lentiCRISPRv2 vector inserted with sgRNA, the packaging plasmid psPAX2 (Addgene, Catalog #12260) and the envelope plasmid VSV-G (Addgene, Catalog #8454) were mixed together and then added to a mixture of Lipofectamine 3000 (Thermo Fisher Scientific) with Opti-MEM (Thermo Fisher Scientific). This transfection mix was kept at room temperature for 30 min (min) and then carefully added into HEK-293T cells in a 60 mm cell culture dish. After 18 h (37 °C, 5% CO_2_), the medium was replaced with growth medium supplemented with 20% FBS, and lentiviruses were harvested at 24 h after changing the medium and then daily for 2 days. The virus-containing media were pooled and centrifuged at 800× *g* for 5 min to remove cell debris. The supernatant was used to infect APRE-19 cells supplemented with 8 μg/mL polybrene (Sigma). The infected cells were selected in media containing puromycin (Sigma) (4 μg/mL), and the resulting cells examined by Western blotting analysis [34,35].

### 2.5. Western Blotting

Detailed protocols were described in our previous report [34]. Briefly, when cultures reached 90% confluence in 24-well plates, they were serum-starved overnight followed by treatment with TGF-β2 (10 ng/mL) for 1–72 h. After being rinsed twice with ice-cold phosphate-buffered saline (PBS), cell lysates were harvested by sample buffer diluted with extraction buffer (10 mM Tris-HCl, pH 7.4, 5 mM EDTA, 50 mM NaCl, 50 mM NaF, 1% Triton X-100, 20 μg/mL aprotinin, 2 mM Na_3_VO_4_ and 1 mM phenylmethylsulfonyl fluoride: PMSF) from 5 × protein sample buffer ((25 mM EDTA (pH 7.0), 10% sodium dodecyl sulfate (SDS) (Sigma-Aldrich Corp., St. Louis, MI, USA), 500 mM dithiothreitol, 50% sucrose, 500 mM TrisHCl (pH 6.8) and 0.5% bromophenol blue)). Samples were then boiled for 5 min, centrifuged for 5 min at 13,000× *g*, separated by 10% SDS-PAGE, transferred to polyvinylidene difluoride membranes and subjected to Western blot analysis. Experiments were repeated at least 3 times. Signal intensity was determined by densitometry with ImageJ software.

### 2.6. Immunofluorescence

Immunofluorescence was performed as described previously [34,35]. Briefly, following starvation and treatment with TGF-β2, cells were fixed in 3.7% formaldehyde/PBS for 10 min at room temperature, followed by blocking with 5% normal goat serum in 0.3% Triton X-100/PBS for 30 min and overnight incubation at 4 °C with primary antibodies (1:200 dilution). After thorough washes with 0.3% Triton X-100/PBS to remove non-specific binding, samples were incubated with fluorescent-labeled secondary antibodies Dylight 549 or 488 (anti-rabbit IgG) (1:300 dilution in a blocking buffer) for 1 h, followed by washing with 0.3% Triton X-100/PBS, mounting in mount medium with 4′,6-diamidino-2-phenylindole (DAPI) (Vector Laboratories) and photographing using a fluorescent microscope. Experiments were repeated at least 3 times.

### 2.7. Quantitative PCR

Total RNA of TGF-β2-treated ARPE-19 cells was extracted using the RNeasy Plus Mini Kit (QIAGEN, Germantown, MD, USA). Primers for quantitative PCR were synthesized by Integrated DNA Technology (Coralville, IA, USA) with the following sequences: forward: 5′-TGAAGCAGCCATGGCAGAAGTA-3′, reverse: 5′-TCCTGGAAGGAGCACTTCAT CT-3′ for human *IL-1β*; forward: 5′-GGAGAAGTTTTTGAAGAGGGCTG-3′, reverse: 5′-ACAGACCCACACAATACATGAAG-3′ for human *IL-8*; forward: 5′-GAGAAGA TTCCAAAGATGTA-3′, reverse: 5′-TTACTCTTGTTACATGTCTC-3′ for human *IL-6*; forward: 5′-AATGGGCAGCCGTTAGGAAA-3′, reverse: 5′-GCCCAATACGACCAA ATCAGAG-3′ for the housekeeping gene *GADPH*.

### 2.8. Cell Migration Assay

This assay was conducted according to procedures in our published literature [34]. Briefly, confluent monolayers of cells in 24-well plates were scratched using autoclaved 200 μL pipet tips across the wells and detached cells aspirated away using PBS. The remaining cells were treated with TGF-β2 (10 ng/mL). Scratched areas were photographed once after scratching for the initial width and again 18 h later. The data were analyzed using Image J and Adobe Photoshop CS4 software as described previously [34]. At least 3 independent experiments were performed.

### 2.9. Luciferase Assay-PIK3CD Reporter

This assay was conducted according to the Promega Luciferase Assay Systems. Cells seeded into 96-well plates were used at around 90% confluence. PGL3-*PIK3CD* (the p110δ gene) reporter vectors *p-GL3-R1*, *p-GL3-GN43* (or *p-GL3 control*) and *pRL-RK* were transfected overnight into ARPE-19 cells with lipofectamine 3000 (Life Technologies), followed by treatment with TGF-β2 for 24 h. After washing with PBS twice, cells were lysed with 1× passive lysis buffer, gently rocked for 15 min at room temperature, followed by addition of 50 μL LAR (luciferase reporter assay) to a 12 mm × 50 mm tube (Part #E2371, Promega. Disposable Cubettes), and the lysates were transferred from a well into the LAR substrate. These mixtures were first read for firefly luciferase activity in a TD-20/20 luminometer (Turner Designer, Promega). Finally, 20 μL 1× Stop and Glo substrate was added into the tubes, mixed and read again to obtain Renilla luciferase activity [28]. At least 3 independent experiments were performed.

### 2.10. Chromatin Immunoprecipitation Assay

Chromatin immunoprecipitation (ChIP) was conducted as described previously (35). In brief, cross-linking of protein-DNA complexes was performed by adding 37% paraformaldehyde diluted to a 1% final concentration and incubation of cells at room temperature for 15 min, followed by addition of glycine (125 mM) to quench the fixation. In total, 500 μL of lysis buffer (10 μg/mL leupeptin, 10 μg/mL of aprotinin and 1 mM PMSF were added per 5 × 10^6^ cells for resuspension. Cell lysates were sonicated to shear chromatin to an average length of ~1 kb, followed by supernatant collected after centrifugation at 12,000× *g*. Agarose beads were incubated with 5 μg of an anti-NFκB or non-immune rabbit IgG at 4 °C with a rotation for 2 h, followed by adding samples to the beads and incubation at 4 °C overnight. The following day, beads were collected by centrifugation and washed 4 times. In total, 100 μL of Tris-EDTA buffer was added to the sample and boiled for 10 min. Finally, samples were centrifuged for 1 min at 12,000× *g*, and the supernatant was collected into a clean tube. The ChIP samples were amplified by PCR using the following primers: forward: 5′-AAGGAGGGAGAGATGGGA-3′, reverse: 5′-ATACACGCGCTCGCTCTT-3′ for *PIK3CD*-2e promoter, and they were subjected to a gel analysis to detect NFκB-bound DNA.

### 2.11. Statistics

Data from 3 independent experiments were analyzed using an unpaired t-test between two groups and one-way analysis of variance (ANOVA) followed by the Tukey honest significant difference (HSD) post hoc test among more than two groups as described preciously [34]. A *p* value less than 0.05 was considered significant difference.

## 3. Results

### 3.1. RNA Sequencing Analysis Uncovers TGF-β2-Induced PI3K/Akt Signaling in RPE Cells

**Figure 1:**
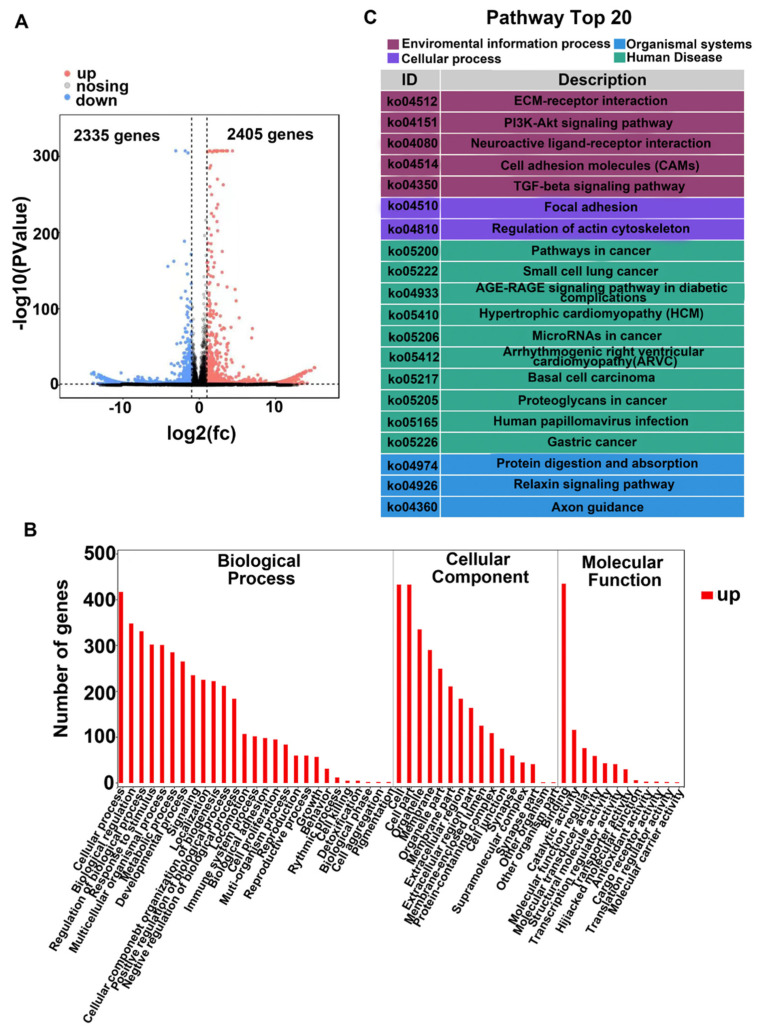
RNA sequencing analysis of TGF-β2-induced major changes in human RPE cells. (**A**). Number of significantly altered genes in TGF-β2-treated ARPE-19 cells. Volcano plot: *X*-axis: indication of the protein difference multiple (take log2); *Y*-axis: corresponding −log10 (*p* value). Red points: significantly up-regulated proteins; green points: significantly down-regulated proteins; gray points: proteins without significant changes. Untreated cells were used as controls. (**B**). GO analysis of biological processes, cellular components and molecular functions of genes altered by TGF-β2 treatment. (**C**). KEGG enrichment of signaling pathways in TGF-β2-treated cells. Top 20 of up-regulated pathways.

The levels of TGF-β2 in the vitreous are known to be associated with PVR [36], with TGF-β2 being considered to be the primary factor to induce EMT [5,25,36]. In order to identify molecules involved in EMT induced by TGF-β2 in a human RPE cell line (referred to as ARPE-19), we performed transcriptomic sequencing analysis. The results showed 4740 differentially regulated genes, of which 2405 were up-regulated and 2335 down-regulated (Figure 1A).

Gene ontology (GO) analysis of these genes sets indicated a broad distribution of these TGF-β2 targets in the intracellular organelle and membranes (Figure 1B), with biological processes connected with cellular responses such as cell adhesion, immune system process and cell proliferation. Molecular function analysis indicated that TGF-β2 induced binding, structural and transcription regulator activities (Figure 1B).

Kyoto Encyclopedia of Genes and Genomes (KEGG) analysis predicted that TGF-β2 regulated processes related to focal adhesion and the actin cytoskeleton as well as the activated PI3K/AKT signaling pathway (Figure 1C), as well as immune system processes and transcription regulator activities. Based on these observations, we speculated that the nuclear transcription factor (NFκB), which is key in inflammatory and immune responses [37], also played a role in experimental PVR [27,28,29].

### 3.2. Depletion of NFκB/p65 in RPE Cells Attenuates TGF-β2-Induced Akt Activation, Expression of p110δ as Well as EMT

**Figure 2:**
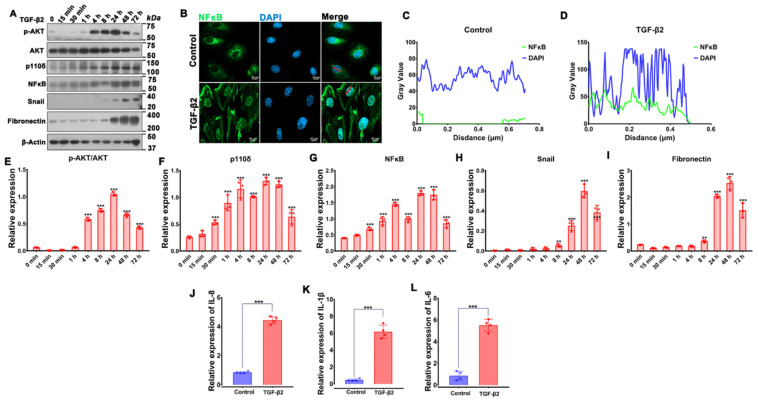
TGF-β2-induced activation of Akt, expression of p110δ, nuclear translocation of NFκB/p65 as well as EMT. (**A**). ARPE-19 cells were treated with TGF-β2 (10 ng/mL) for the indicated time points, followed by immunoblotting analyses. (**B**). ARPE-19 cells treated with TGF-β2 (10 ng/mL) for 48 h were immunofluorescence stained with antibodies to NFκB/p65 (green). Blue: DAPI. Red line: the location of the co-localization. Scale bar: 10 μm. (**C**,**D**). Co-localization analysis of NFκB with nucleus was performed by Image J [40]. (**E**–**I**). Quantitation of Western blotting band intensity in A. The graphs are mean ± standard deviation (SD) of three independent experiments. ** *p* < 0.01, *** *p* < 0.001. (**J**–**L**). mRNA from ARPE-19 cells treated with TGF-β2 (10 ng/mL) for 48 h was extracted and subjected to reverse qPCR. The graphs are mean ± SD of 4 independent experiments. The data were analyzed using one-way ANOVA followed by the Tukey HSD post hoc test. ** *p* < 0.01, *** *p* < 0.001.

It is not clear at present which of the PI3K family members plays a predominant role in TGF-β2-induced EMT in RPE cells. We previously reported that PI3Kδ inactivation prevents vitreous-induced activation of Akt in human RPE cells [34], and other researchers showed that Akt could be activated by TGF-β2 [38,39]. We therefore speculated that PI3Kδ could play a role in TGF-β2-stimulated Akt activation in RPE cells. To gain insight into this, we performed a time course of TGF-β2-induced Akt activation. As shown in Figure 2A,E, TGF-β2 stimulated activation of Akt, as measured by phosphorylation on Ser473 (8.84 ± 1.14 fold) within 4 h, but it decreased at 48 h; in addition, the expressions of snail and fibronectin, markers of EMT, were increased (snail: 44.79 ± 26.93 fold; fibronectin: 8.43 ± 0.21 fold) after 24 h upon TGF-β2 treatment (Figure 2A,H,I).

NFκB/p65 is capable of transcriptionally inducing p110δ expression in vascular ECs [30]. Hence, we next examined whether TGF-β2 was able to enhance expression of p110δ and NFκB/p65. Western blot analyses confirmed that expression of p110δ and NFκB/p65 was elevated (3.72 ± 1.13 fold) and (2.95 ± 1.09 fold), respectively, in ARPE-19 cells treated with TGF-β2 for 24 h (Figure 2A,F,G).

Based on the notion that TNFα induces NFκB/p65-dependent expression of p110δ [30], we explored whether TGF-β2 could also stimulate p110δ transcription via NFκB/p65. Firstly, we examined the cellular localization of p65 as a marker of NFκB activation after TGF-β2 treatment. Immunofluorescence analyses confirmed that TGF-β2 treatment could promote the nuclear transfer of NFκB (Figure 2B–D). Expression of NFκB-dependent genes (e.g., IL-8, IL-1β and IL-6) was also up-regulated by TGF-β2 treatment (Figure 2J–L), suggesting that TGF-β2 is able to activate NFκB.

**Figure 3:**
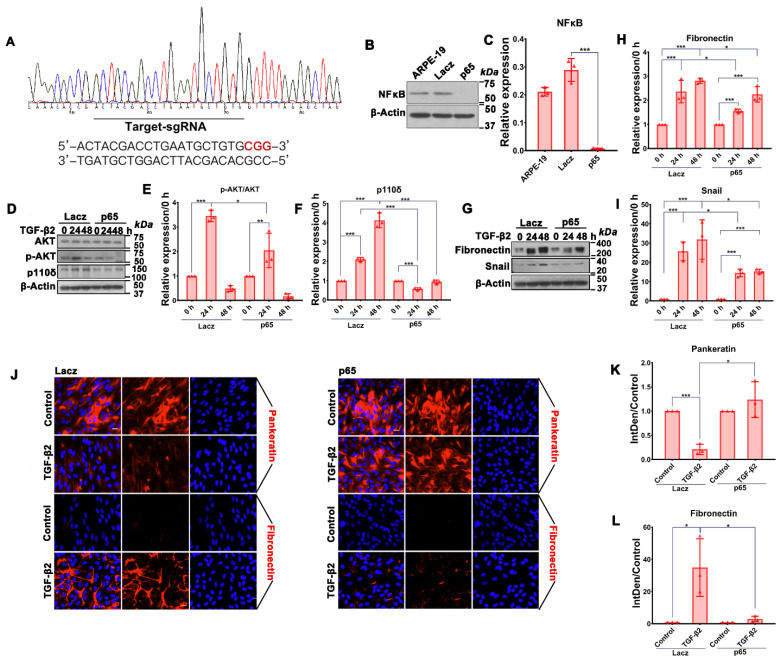
Depletion of NFκB/p65 attenuates TGF-β2-induced Akt activation, expression of p110δ as well as EMT. (**A**). Sanger DNA sequencing identification of CRISPR-Cas9-mediated genome editing. (**B**). Western blotting analysis identification of CRISPR-Cas9-mediated protein depletion using the indicated antibodies. Shown is a representative of at least 3 independent experiments. (**C**). Quantitation of Western blotting band intensity. The graphs are mean ± SD of three independent experiments. The data were analyzed using one-way ANOVA followed by the Tukey HSD post hoc test. (**D**,**G**). Serum-starved cells with *LacZ* or NF-κB/p65 sgRNA were treated with TGF-β2 (10 ng/mL) for 24 h or 48 h. Cell lysates were subjected to immunoblotting analysis using the indicated antibodies. Shown is a representative of at least 3 independent experiments. (**E**,**F**,**H**,**I**). Quantitation of Western blotting band intensity in (**D**,**G**). The bar graphs are mean ± SD of 3 independent experiments. The data were analyzed using one-way ANOVA followed by the Tukey HSD post hoc test. (**J**). ARPE-19 cells expressing SpCas9 with *LacZ* or *RELA* (encoding NF-κB/p65) sgRNA treated with TGF-β2 (10 ng/mL) for 48 h were stained with antibodies to fibronectin (rabbit) and pan-keratin (mouse), followed by incubation with fluorescently labeled secondary antibodies. The slides were mounted in mounting medium containing DAPI (blue). Red signals indicate protein expressions. Shown is a representative of three independent experiments. Scale bar: 20 μm. (**K**,**L**). Integrated density of the fluorescence was analyzed by Image J. The graphs are mean ± SD of 3 independent experiments. The data were analyzed using one-way ANOVA followed by the Tukey HSD post hoc test. * *p* < 0.05, ** *p* < 0.01, *** *p* < 0.001.

To determine if TGF-β2-induced expression of p110δ was dependent on NFκB, we established an ARPE-19 cell line with NFκB/p65 depleted by CRISPR/Cas9 using a sgRNA targeting *RELA* encoding NFκB/p65 (Figure 3A). As shown in Figure 3B,C, there was a more than 90% reduction in NFκB/p65 expression in the NFκB/p65-depleted cells compared with those expressing *lacZ*-sgRNA as a control. Intriguingly, upon on the depletion of NFκB/p65, there was concomitance with an attenuation of TGF-β2-induced Akt activation and p110δ expression (Figure 3D–F). These results encouraged us to investigate if depletion of NFκB/p65 could suppress TGF-β2-induced EMT of ARPE-19 cells. Both the Western blot and immunofluorescence analyses showed that depletion of NFκB/p65 blocked TGF-β2-induced expression of fibronectin and snail markers for EMT (Figure 3G–L).

### 3.3. Depletion of PI3Kδ Blocks TGF-β2-Induced Akt Activation and NFκB/p65 Protein Expression in RPE Cells

**Figure 4:**
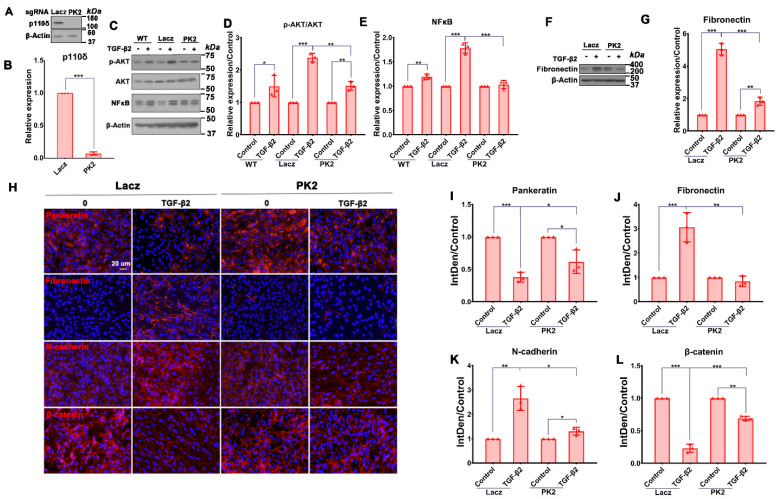
Depletion of NFκB/p65 attenuates TGF-β2-induced Akt activation, p110δ expression and EMT. (**A**,**B**). ARPE-19 cells with lacZ-sgRNA/SpCas9 or PK2-sgRNA/SpCas9 were examined by Western blotting using indicated antibodies. Shown is a representative of at least 3 independent experiments (**A**); Quantitation of Western blotting band intensity in A (**B**). (**C**–**G**). Serum-starved ARPE-19 expressing SpCas9 with LacZ or PK2 sgRNA were treated with TGF-β2 (10 ng/mL) for 48 h. Their lysates were subjected to Western blot analyses using indicated antibodies. Shown is representative of at least 3 independent experiments (**C**,**F**). Quantitation of Western blotting band intensity in (**C**–**G**). (**H**). ARPE-19 cells expressing SpCas9 with LacZ or PK2 sgRNA treated with TGF-β2 (10 ng/mL) for 48 h were first stained with antibodies against fibronectin (rabbit), pan-keratin (mouse), N-cadherin (rabbit) and β-catenin (rabbit), followed by incubation with fluorescently labeled secondary antibodies and mounting of the slides in mounting medium containing DAPI (blue). Red signals indicate proteins expression. Shown is a representative of 3 independent experiments. Scale bar: 20 μm. (**I**–**L**). Integrated density of the fluorescence was analyzed by Image J. The bar graphs are mean ± SD of 3 independent experiments. * *p* < 0.05, ** *p* < 0.01, *** *p* < 0.001.

PI3Kδ is essential in vitreous-induced activation of Akt, which can activate NFκB. Thus, we reasoned that TGF-β2 might induce a PI3Kδ/Akt/NFκB/PI3Kδ signaling loop in RPE cells. Indeed, Western blot analyses showed that depletion (93.00 ± 2.65%) of p110δ expression by CRISPR/Cas9 in ARPE-19 cells (Figure 4A,B) prevented TGF-β2-induced Akt activation and NFκB/p65 expression (Figure 4C–E). These data suggested to us that TGF-β2 induces a signaling loop of PI3Kδ/Akt/NFκB/PI3Kδ and that breaking this signaling loop may prevent TGF-β2-induced EMT, a key cellular event in PVR pathogenesis [25,41,42].

### 3.4. Depletion of PI3Kδ in RPE Cells Prevents TGF-β2-Induced EMT

ARPE-19 cells undergo EMT upon stimulation with TGF-β2 [35], and these cells can also induce experimental PVR [43]. Hence, we next examined if PI3Kδ depletion in ARPE-19 cells could block TGF-β2-induced expression of fibronectin, a protein marker of EMT. As previously reported [35], TGF-β2 induced expression of fibronectin in ARPE-19 cells, with this expression being prevented (73.8 ± 2.3%) by CRISPR/Cas9-mediated depletion of p110δ as shown by analyses of Western blotting (Figure 4F,G) and immunofluorescence (Figure 4H–L). Taken together, depletion of PI3Kδ in ARPE-19 cells is able to block TGF-β2-induced EMT.

### 3.5. NFκB/p65 Binding to the PIK3CD-2e Promoter Is Required for the TGF-β2-Induced p110δ Expression in RPE Cells

**Figure 5:**
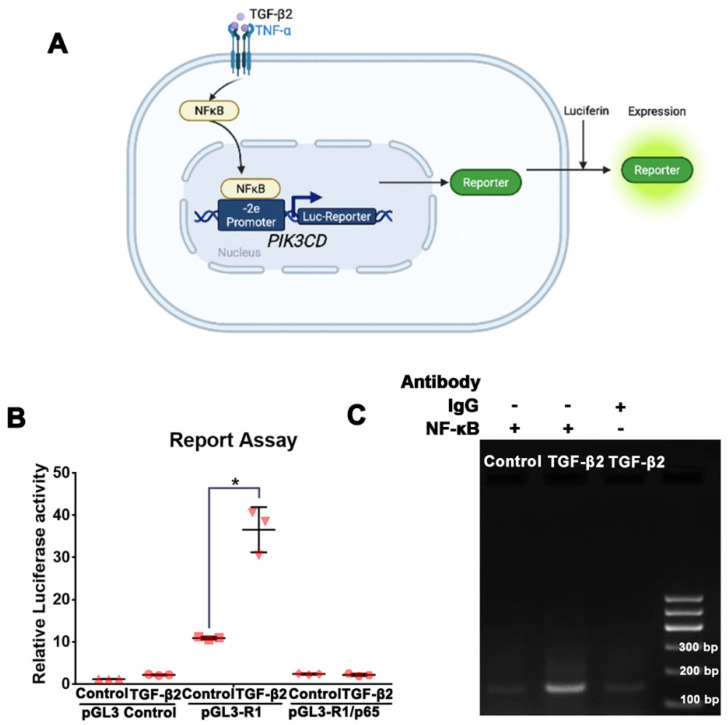
NFκB/p65 binding to the *PIK3CD*-2e promoter is required for TGF-β2-induced expression of p110δ in ARPE-19 cells. (**A**,**B**). After cells had attached to a 96-well plate, they were transfected overnight with p-GL3-R1 (or p-GL3 control) or pRL-RK using lipofectamine 3000. Cells were then treated with TGF-β2 (10 ng/mL) for the next 24 h. Firefly luciferase activity and Renilla luciferase activity were read in a TD-20/20 luminometer. Data were calculated as firefly luciferase activity/Renilla luciferase activity. The mean ± SD of 3 independent experiments is shown. Data were analyzed using one-way ANOVA followed by the Tukey HSD post hoc test. * Denotes *p* < 0.05. (**C**). NFκB/p65 binding to the PIK3CD-2e promoter determined by a ChIP assay. DNA from ChIP was subjected to PCR and gel analysis. Non-immune IgG served as a negative control.

To gain additional insight into the mechanism of p110δ expression, we next evaluated if the PIK3CD promoter element responsible for the TGF-β2 responsiveness was the same locus as previously shown for TNFα [30]. To this end, we transfected ARPE-19 cells with a pGL3-basic luciferase reporter plasmid or a pGL3 plasmid containing the PIK3CD (-2e) promoter element, which contains an NFκB/p65 binding region that is responsive to TNFα stimulation in human ECs [30] and then treated these cells with TGF-β2 for 24 h (Figure 5A). A luciferase reporter assay showed that the basal activity of the exon-2e promoter [30] was comparable in cells with or without stimulation, but its activity was enhanced upon TGF-β2 simulation; furthermore, depletion of NFκB/p65 diminished the TGF-β2-induced binding of this TF to the exon-2e promoter in ARPE-19 cells (Figure 5B). Furthermore, ChIP using an antibody against NFκB/p65 showed that NFκB could bind to the PIK3CD-2e promoter (primers designed based on the sequences of PIK3CD-2e promoter) in the ARPE-19 cells treated with TGF-β2 (Figure 5C). Taken together, these results demonstrate that NFκB/p65 binding to the PIK3CD-2e promoter is required for TGF-β2-induced expression of p110δ in ARPE-19 cells, similar to as was shown for response to TNFα stimulation in vascular ECs.

### 3.6. Depletion of PI3Kδ Prevents TGF-β2-Induced Migration of RPE Cells

**Figure 6:**
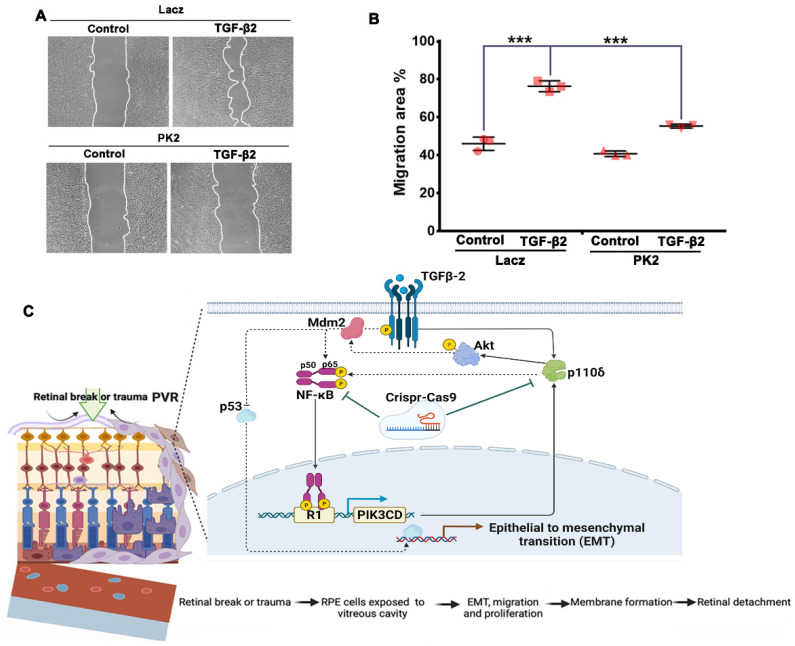
Depletion of PI3Kδ blocks TGF-β2-induced migration of ARPE-19 cells. (**A**). Wells of confluence cells were scratched using 200 μL pipet tips, washed and photographed at the initial width, followed by treatment with TGF-β2 (10 ng/mL) for 24 h. Cell images were then taken, and wound areas were analyzed by image J and Adobe Photoshop CS4 software. The data of bar graphs are the mean ± SD of 3 independent experiments. Representative raw data of 3 independent experiments are shown underneath the bar graphs. (**B**). *** denotes *p* < 0.001 using one-way ANOVA followed by the Tukey HSD post hoc test. (**C**). Diagram of a loop pathway of PI3Kδ/Akt/NFκB/PI3Kδ induced by TGF-β2. Growth factors (GFs) and cytokines (CKs) in the vitreous [24,45] activate the PI3K/Akt signaling pathway, resulting in Mdm2 phosphorylation and a decline in p53 levels [28,44]. Receptor-regulated PI3Ks consist of PI3Kα, PI3Kβ and PI3Kδ [31]. In particular, TGF-β2 in the vitreous [46] triggers heightened expression of Mdm2 [47], resulting in elevated levels of NFκB/p65 [48] and p110δ [30], the catalytic subunit of PI3Kδ [31]. These biochemical events (e.g., a decrease in p53 [49,50,51] and an increase in NFκB/p65 [52,53]) consequently promote cellular responses (e.g., proliferation, EMT, migration and contraction), driving PVR pathogenesis.

We next used a scratch-wound assay to examine if PI3Kδ depletion could prevent TGF-β2-induced migration of ARPE-19 cells because this cellular event is critical for the formation of epiretinal membranes, whose contraction causes retinal detachment [44]. As shown in Figure 6A,B, TGF-β2 promoted migration of ARPE-19 cells with lacZ-sgRNA/SpCas9 at 16 h, but depletion of p110δ with PK2-sgRNA/SpCas9 significantly attenuated TGF-β2-induced migration of these cells at this time point, demonstrating that inactivation of PI3Kδ could prevent PVR pathogenesis.

## 4. Discussion

In the present study, to explore the molecular mechanism by which EMT develops in RPE cells, RNA sequencing was employed to profile TGF-β2-induced changes in cultured RPE cells. These studies identified the PI3K/AKT signaling pathway to be significantly up-regulated upon the TGF-β2 treatment. We previously showed that PI3Kδ plays an essential role in vitreous-induced Akt activation, and another research team demonstrated that TGF-β2 levels in vitreous from patients with PVR are associated with clinical PVR [28,54]. In the current study, we found that PI3Kδ is critical for TGF-β2-induced Akt and cellular responses including EMT and migration of RPE cells. At this stage, it remains elusive whether other PI3K isoforms are involved in PVR pathogenesis and how TGF-β2 may selectively use PI3Kδ to activate Akt. Indeed, other PI3K isoforms are also present in RPE cells [34], and it is not clear why they can apparently not compensate for PI3Kδ inactivation. PI3K isoforms have been shown to have isoform-selective functions at the cellular and organismal level, which may relate to their relative expression levels and isoform-selective coupling to upstream activating signals such as small GTPases [55]. PI3Kδ is highly expressed in human REP cells (34), and thereby the existence of a TGF-β2-induced PI3Kδ/Akt/NFκB/PI3Kδ feedback loop (Figure 6C) to enhance PVR pathogenesis may contribute to such a PI3K isoform-specific function.

We recently showed that PI3Kδ is also important in vitreous-induced activation of Mdm2 [34]. Other studies revealed that patients who harbor the MDM2 single nucleotide polymorphism (SNP) 309 G allele (MDM2^SNP309G^) are more likely to develop PVR [56] and that primary human fetal RPEs containing MDM2^SNP309G^ have greater potential to induce PVR in an animal model [21,57]. The MDM2 gene contains two promoters designated P1 and P2 [58,59], with P1 being considered as a housekeeping promoter, while P2 at the intron between exons 1 and 2 can be activated by a variety of transcription factors including the small mothers against decapentaplegic (Smad)3/4 complex [47] and specificity protein (Sp)1 in response to various stimuli such as TGF-β2 [58]. Blockade of the P2-driven MDM2 expression prevents vitreous-induced p53 degradation (49] or TGF-β2-induced EMT [35]. In addition, Mdm2 also executes activities independently of p53, including induction of nuclear factor (NF)κB subunit p65 (NFκB/p65) transcription [60]. Thus, the major function of the TGF-β2-induced loop of PI3Kδ/Akt/NFκB/PI3Kδ might be to suppress the levels of p53 via Mdm2 because p53 is a gatekeeper of retinal detachment [44]. As a matter of fact, both a decrease in p53 [34,44] and an increase in NFκB/p65 [46] can promote inflammation contributing to PVR pathogenesis (e.g., EMT).

In this study, we also found that depletion of p110δ significantly suppressed NFκB/p65 expression (Figure 4A). Based on that, Mdm2 is able to transcriptionally induce NFκB/p65 expression [60], and we hypothesized that PI3Kδ affected NFκB abundance via Mdm2, which was activated by TGF-β2-induced PI3Kδ/Akt signaling. Thereby, we proposed that a circuit of PI3Kδ/Akt/Mdm2/NFκB/PI3Kδ triggered by TGF-β2 might exist in RPE cells. This feedback loop is currently under investigation.

PVR is still a major obstacle to successfully correct RD despite gradual improvements in surgical success rates over the past decades; in particular, there are over 75% of postsurgical re-detachments [61]. However, there is still no effective treatment for this blinding disease even though numerous clinically approved anti-proliferative medicines have been evaluated [61,62]. Nonetheless, novel approaches including CRISPR/Cas9 have shed light in clinical trials on the treatment of human diseases, especially eye diseases (62). In summary, based on our discovery of the molecular mechanism of PVR pathogenesis (e.g., EMT) [34,63], a genome-editing approach to targeting the PI3Kδ/Akt/NFκB/PI3Kδ feedback loop (Figure 6C) has great potential for therapeutic treatment of EMT-related diseases, including PVR, addressing a currently unmet clinical need [64].

## Data Availability

The materials described in this report, including all relevant raw data, will be freely available to any researcher wishing to use them for noncommercial purposes, without breaching participant confidentiality.
